# Frailty Markers and Treatment Decisions in Patients Seen in Oncogeriatric Clinics: Results from the ASRO Pilot Study

**DOI:** 10.1371/journal.pone.0149732

**Published:** 2016-02-26

**Authors:** Anaïs Farcet, Laure de Decker, Vanessa Pauly, Frédérique Rousseau, Howard Bergman, Catherine Molines, Frédérique Retornaz

**Affiliations:** 1 Unité de coordination en oncologie gériatrique, Centre Gérontologique Départemental, Marseille, France; 2 Département de Gériatrie, Centre Hospitalo-Universitaire, Nantes, France; 3 Aix Marseille Université, Laboratoire de Santé Publique EA 3279, Faculté de médecine Centre d’Evaluation de la Pharmacodépendance-Addictovigilance (CEIP-A) de Marseille (PACA-Corse) Associé, Marseille, France; 4 Unité de coordination en oncologie gériatrique, Institut Paoli Calmette, Marseille, France; 5 Department of Family Medicine, Mcgill University, Montréal, QC H3T 1E2, Canada; 6 Unité de soins et de recherche en médecine interne et maladies infectieuses, Hôpital Européen, Marseille, France; University of Naples Federico II, ITALY

## Abstract

**Background:**

Comprehensive Geriatric Assessment (CGA) is the gold standard to help oncologists select the best cancer treatment for their older patients. Some authors have suggested that the concept of frailty could be a more useful approach in this population. We investigated whether frailty markers are associated with treatment recommendations in an oncogeriatric clinic.

**Methods:**

This prospective study included 70 years and older patients with solid tumors and referred for an oncogeriatric assessment. The CGA included nine domains: autonomy, comorbidities, medication, cognition, nutrition, mood, neurosensory deficits, falls, and social status. Five frailty markers were assessed (nutrition, physical activity, energy, mobility, and strength). Patients were categorized as Frail (three or more frailty markers), pre-frail (one or two frailty markers), or not-frail (no frailty marker). Treatment recommendations were classified into two categories: standard treatment with and without any changes and supportive/palliative care. Multiple logistic regression models were used to analyze factors associated with treatment recommendations.

**Results:**

217 patients, mean age 83 years (± Standard deviation (SD) 5.3), were included. In the univariate analysis, number of frailty markers, grip strength, physical activity, mobility, nutrition, energy, autonomy, depression, Eastern Cooperative Oncology Group Scale of Performance Status (ECOG-PS), and falls were significantly associated with final treatment recommendations. In the multivariate analysis, the number of frailty markers and basic Activities of Daily Living (ADL) were significantly associated with final treatment recommendations (p<0.001 and p = 0.010, respectively).

**Conclusion:**

Frailty markers are associated with final treatment recommendations in older cancer patients. Longitudinal studies are warranted to better determine their use in a geriatric oncology setting.

## Introduction

Both the incidence of cancer and the risk of death due to cancer increase with age [1]. Demographic projections for 2030 suggest that people older than 65 will represent almost 25% of the European population, and death by cancer will represent the first cause of mortality. The elderly population is heterogeneous in terms of health problems such as comorbidities, disabilities, polymedication, cognition, mood, social issues, etc. Management of cancer in the elderly population is challenging because of potential underlying health problems that may interfere with treatment. The International Society of Geriatric Oncology (SIOG) [2], and several literature reviews recommend an approach based on comprehensive geriatric assessments (CGA) to help specialists in selecting the best cancer treatment [1,3,4,5,6].

Balducci’s and colleagues proposed recommendation for treatment plan based on CGA results. Three groups were defined: vulnerable, intermediate and palliative. For the intermediate group, decision could be either palliative of curative. Literature reviews have recently questioned decision making strategies [7,8,9]. In studies that examined the impact of CGA in treatment decision-making [10,11,12,13,14] only three [11,12,14] showed that, in 30% to 50% of patients, CGA led to changes in the oncologic treatment plan. Then additional tools could be, therefore, necessary in the older cancer population.

Using data from the Cardiovascular Health Study, Fried and colleagues [15] identified five frailty markers: nutrition, mobility, strength, energy, and physical activity. They reported that older persons with at least three of the five frailty markers are at a significantly increased risk of suffering from adverse outcomes such as falls, worsening mobility, disability, hospitalization, and death within three years. Moreover, the presence of at least one of these markers confers an higher risk of adverse outcomes [15,16]. In oncology setting, presence of frailty markers predicts treatment toxicity and risk of early death [17]. In oncology surgery setting, frailty markers are associated with an higher risk for postoperative complications [18,19] and length of stay[18]. Thus, the concept of frailty could be a useful approach to detect potential underlying health problems that may interfere with treatment in older cancer patients [20].

The aim of this study was to assess whether frailty markers and CGA are associated with cancer treatment recommendations in an oncogeriatric clinic.

## Materials and Methods

### Ethics Statement

Informed consent was not obtained from the patients. The database was anonymous. The Ethics Comittee of the Centre gérontologique départemental of Marseille, France, approved the study and its protocol. The french data protection agency (Commission Nationale de l'Informatique et des Libertés–CNIL) approved the use of the database for clinic research (record number: 1641373 v.0. January 2, 2013).

### Study Setting, Sample, and Design

This cross-sectional descriptive study was carried out for the purpose of oncogeriatric assessment by a single team between January 2008 and June 2013 in three centers of Marseille. The team included two trained geriatricians in oncology, nurse practitioners, dietitians, a social worker, and pharmacists.

Inclusion criteria for this study were: 70 years and older patients, having a solid tumor and referred by their physician to the oncogeriatric clinics after an initial cancer treatment plan. Patient with life expectancy estimated to be 3 month and/or previously treated with chemotherapy or radiotherapy, or patient seen for follow-up, were excluded from the study.

**Fig 1:**
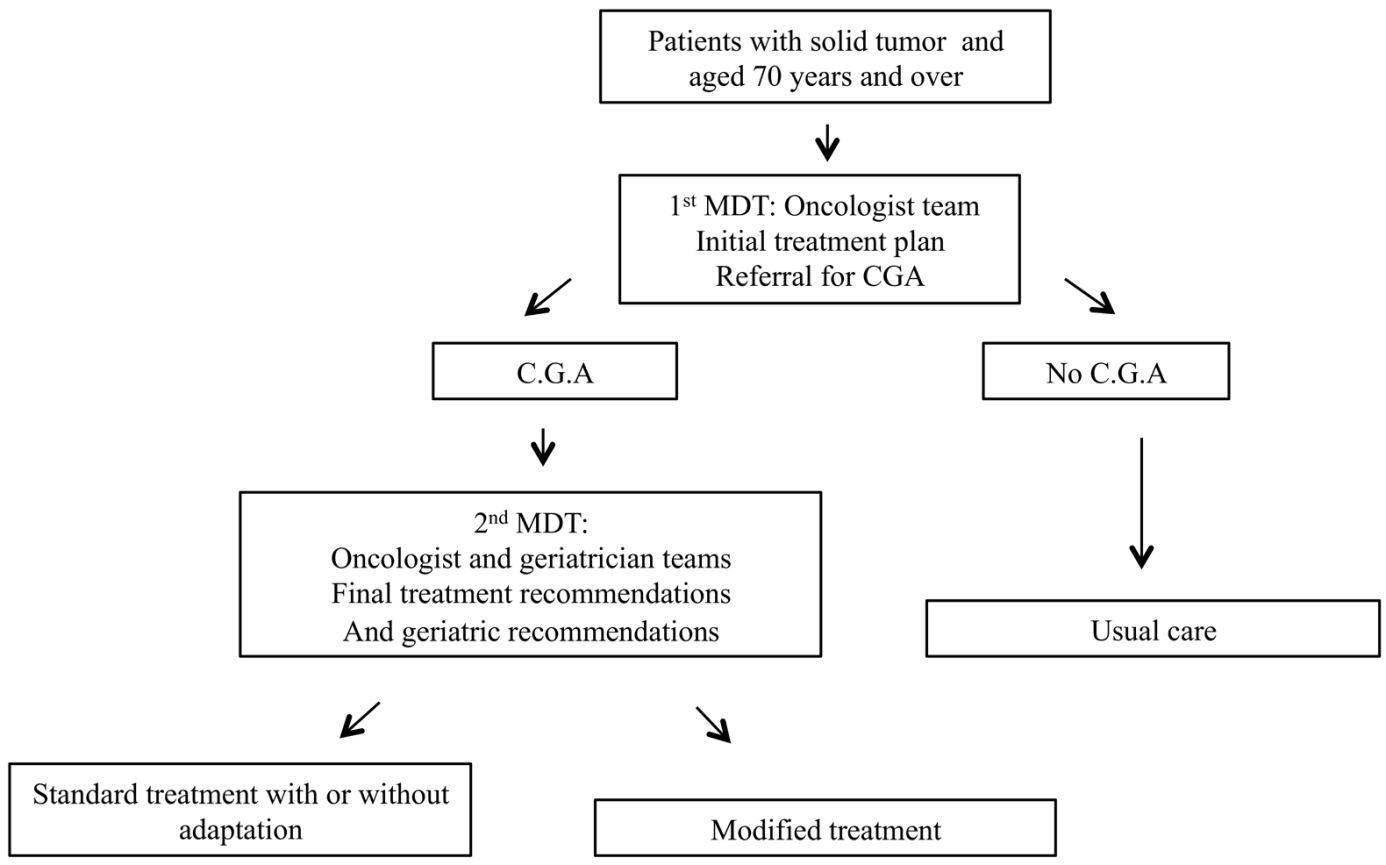
Patients seen in oncogeriatric clinics.

The following procedure was applied (Fig 1). Older cancer patients were referred to oncologist or surgeon for cancer assessment. Initial cancer treatment plan was discussed during a 1^st^ multidisciplinary team meeting (MDT). After MDT, an initial cancer treatment plan was decided (surgery, radiotherapy, chemotherapy, supportive/palliative care) requiring or not CGA. After CGA, oncologist and geriatricians met during a 2^nd^ MDT to decide final treatment recommendations.

Geriatric recommendations might be proposed depending on the geriatric syndrome identified.

### Data Collection

Age, sex, and Eastern Cooperative Oncology Group Scale of Performance Status (ECOG-PS) [21] were recorded by nurse practitioner. Oncology data, including type and stage of cancer; treatment cancer plan and reason for assessment were collected by the geriatrician. CGA data and frailty markers were collected by both the geriatrician and the nurse practitioner.

The CGA used both self-report and performance-based measures. The CGA included nine domains: functional status, comorbidities, medication, cognition, nutrition, mood, neurosensory deficits, falls, and social status. The functional status was assessed using activities of daily living (ADL), instrumental activities of daily living (IADL), and the Eastern Cooperative Oncology Group Scale of Performance Status (ECOG-PS). ADL disability was assessed using six tasks of the Katz index [22]. IADL disability was assessed using the seven Older American Resources and Services (OARS) items [23]. The denominator was adjusted to take into account patients who did not normally perform an activity such as cooking or doing laundry. Disability in ADL or IADL was defined as the need for assistance to complete at least one ADL or IADL, respectively. The comorbid conditions were codified according to the International Coding Diseases (ICD-10th revision, French version). Ten groups of comorbidities were selected: cardiovascular disease, hypertension, diabetes, depression, dementia, other neurological diseases, respiratory disease, gastrointestinal disease, osteoarticular disease, and renal failure [24]. In each group, patients scored positive if they had one or more comorbidities. Burden comorbidity was defined by the presence of three or more comorbidities [25]. The number of medications (excluding those for cancer treatment) was calculated for each patient. The cognition was assessed by the following tests: the Mini-Mental State Examination [26] (MMSE), the Mini Cog[27], the Montreal Cognitive Assessment (MoCA) [28], or the Clock test [29]. Cognitive disorders were defined by MMSE or MoCA < 26 and/or a pathologic Mini-COG or Clock test. The 4-item Geriatric Depression Scale (mini GDS) was used to screen for depression. A score of 1 or more indicated depression [30]. Patients with trouble hearing and/or requiring hearing aids, and/or patients with trouble seeing (despite the use of glasses) were considered to have a neurosensory deficit. Patients who had experienced one or more falls in the previous six months were considered to have a positive history of falls. The nutritional status was assessed by body mass index (BMI). A BMI under 22 was considered to be underweight and indicated under-nutrition [31]. As no gold standard to evaluate social support and home care services was available, the following questions were asked: “If necessary, do you have someone who can care for you? If yes, who is it? Do you have professional help at home? If yes, what kind? Do you have family support or home care services?”

All patients were classified into the three Balducci groups [32]: Group I: patients who are functionally independent for ADL and without serious comorbidity, Group II: patients who are independent for ADL and/or have 1 to 2 comorbidities and/or no geriatric syndrome, Group III: age ≥ 85 years, patients who are dependent for at least one ADL and/or have 3 or more comorbidities and/or at least 1 geriatric syndrome.

The five frailty markers adapted from the Fried phenotype were also recorded: nutrition, energy, strength, physical activity, and mobility [15]. The nutritional status was assessed by two self-report questions: “In the last year, have you lost more than ten pounds unintentionally? [15]. In the last three months, has food intake decreased for whatever reason?” [33]. An affirmative answer to one of the two questions indicated a positive marker of frailty for nutrition. The energy was assessed using a visual scale ranging from 0 (no energy) to 10 (full of energy). A score < 3 indicated a positive marker of frailty for energy [16]. The strength was assessed by three measurements of grip strength (in kilograms) in the dominant hand using a Jamar handheld dynamometer. The maximal grip strength was selected for the analysis. The lowest quintile by sex and BMI was considered a positive marker of frailty for strength [15]. The physical activity was assessed by a validated self-report question from the Canadian Study of Health and Aging Risk Factor Questionnaire (RFQ) [34]. No exercise or a low level of exercise was considered to be a positive marker of frailty for physical activity. The mobility was assessed by the Timed Up and Go test (TUG) [35] or the one-leg standing balance test [36]. A TUG time of less than ten seconds or the inability of a patient to balance on one leg for more than five seconds was considered to be a positive marker of frailty for mobility. Patients who had three or more markers were classified as *frail*, patients with one or two markers as *pre-frail*, and patients with no markers as *not- frail* [15]. CGA data and frailty markers were collected by geriatricians and nurse practitioners except data for nutrition which were collected by the dietitian.

The final treatment recommendations were classified into two categories: Standard treatment (corresponding to the initial treatment plan, with or without adaptation when surgery, chemotherapy or radiotherapy were recommended), modified treatment (patient for whom supportive / palliative care were recommended after geriatric assessment instead of initial treatment plan included surgery, chemotherapy or radiotherapy)

Geriatric recommendations were proposed in eight domains: nutrition, mobility, usual treatment modifications, cognition, comorbidities, functional status, depression, neurosensory deficit.

### Data Analysis

Descriptive statistics of patient characteristics, and health and functional status measures were calculated. A univariate comparison of the three groups defined by the treatment changes/recommendations was performed using the chi-square test or the Fisher exact test for qualitative variables and the Analysis of variance (ANOVA) or the Kruskal-Wallis non-parametric test for quantitative ones. To analyze independent factors associated with treatment recommendations, a multivariate regression statistical analysis was performed. We entered into the model variables which were associated in the univariate analysis with a p-value<0.20, excluding variables which presented collinearity with other factors. Associations with a p-value<5% were considered significant. All statistical analysis was carried out using statistical software SPSS v17.

## Results

In all, 217 patients, with a mean age of 83 years (± SD 5.3), were included in this study. Women represented 58% (Table 1). Digestive cancer was the most common diagnosis, followed by urogenital cancer (39% and 21%, respectively). More than one-fourth of the patients had metastases. Half were referred before chemotherapy and 39% before surgery. Ninety-five percent of patients lived at home. Fewer than one out of six had more than three comorbidities. Almost half had more than five drugs. About one-third had ADL disability, and two-thirds had IADL disability. Roughly 40% had cognitive disorders and depression. One-fourth had fallen in the last six months. The most prevalent of the frailty markers were mobility (77%), physical activity (65%), and nutrition (61%). Only 7% of patients were non-frail; 40% were frail. According to the Balducci classification, only 2% of patients were fit, and 7% were frail.

**Table 1:** Characteristics of patients (N = 217).

**Characteristics**		**Mean +/- SD; n, %**
**Age (years)**		83.2 ± 5.3
**Female**		125 (57.6%)
**Patients, previously diagnosed, followed at (N = 212)**	Oncology departments	141 (65.0%)
	Geriatric departments	67 (30.9%)
	General/internal medicine	4 (1.8%)
**Tumor (N = 216)**	Digestive	84 (39.3%)
	Urological	46 (21.2%)
	Breast and gynecological	38 (17.5%)
	Lung	25 (11.5%)
	Other	23 (10.6%)
**Presence of metastasis (N = 212)**		61 (28.8%)
**Reason for consultation (N = 214)**	Evaluation before chemotherapy	114 (53.3%)
	Evaluation before surgery	85 (39.2%)
	Other	25 (11.5%)
**Geriatric Assessment**		**n, %**
**Living at home (N = 210)**		201 (95.7%)
**Presence of social support (N = 211)**		184 (87.2%)
**Comorbidities (N = 214)**	3 or more comorbidities	38 (17.8%)
	Cardiovascular	110 (51.4%)
	Hypertension	106 (49.5%)
	Osteoarticular	88 (41.1%)
	Diabetes	51 (23.8%)
	Respiratory	36 (16.8%)
	Depression	25 (11.7%)
	Digestive	24 (11.2%)
	Neurological	23 (10.7%)
	Chronic renal failure	18 (8.4%)
	Dementia	15 (7.0%)
	Previous cancer	54 (25.2%)
**Drugs ≥ 5 (N = 206)**		99 (48.1%)
**Functional status**	Abnormal ADL (N = 204)	82 (38.3%)
	Abnormal IADL (N = 215)	137 (63.7%)
	ECOGS PS >1 (N = 92)	27 (29.3%)
**Cognitive impairment (N = 211)**		84 (39.8%)
**BMI < 22kg/m**^**2**^ **(N = 166)**		39 (23.5%)
**History of Falls (N = 217)**		55 (25.3%)
**Depression (N = 213)**		80 (37.5%)
**Neurosensory deficit**	Visual deficit (N = 204)	80 (39.2%)
	Hearing deficit (N = 203)	91 (44.8%)
**Balducci classification (N = 217)**	Group I (fit)	5 (2.3%)
	Group II (intermediate)	197 (90.8%)
	Group III (frail)	15 (6.9%)
**Frailty markers**		**n, %**
**Mobility (N = 200)**		155 (77.5%)
**Physical activity <3 (N = 197)**		129 (65.5%)
**Nutrition (N = 215)**		131 (60.9%)
**Grip strength (N = 184)**		49 (26.6%)
**Energy <3 (N = 169)**		19 (11.2%)
**0 marker (not-frail)**		15 (6.9%)
**1 or 2 markers (pre-frail)**		110 (50.7%)
**at least 3 markers (frail)**		92 (42.4%)
**Final treatment recommendations (N = 217)**		**n, %**
**Standard treatment**		129 (59.4%)
	*Standard treatment without any change*	27 (12.4%)
	*Standard treatment with adaptation*	102 (47.0%)
**Modified treatment**		88 (40.6%)
**Number of geriatric recommendations per patient**		2.20 ± 1.4
**Geriatric recommendations (N = 215)**		**n, %**
**Nutrition recommendations**		101 (45.9%)
**Mobility recommendations**		91 (41.4%)
**Usual treatment modifications**		65 (29.5%)
**Cognition**		53 (24.1%)
**Comorbidities recommendations**		48 (21.8%)
**Functional status recommendations**		34 (15.5%)
**Depression recommendations**		23 (10.5%)
**Neurosensory recommendations**		8 (3.6%)

Abbreviations: ADL: activities of daily living, IADL: instrumental activities of daily living, BMI: body mass index, ECOG–PS: Eastern Cooperative Oncology Group Scale of Performance Status

After assessment, the number of geriatric recommendations was around 2 per patient. The most prevalent recommendations were nutrition (46%) and mobility (41%). Only 24% of patients needed of cognition recommendation and 11% needed depression recommendation (Table 1).

In the univariate analysis, number of frailty markers, physical activity, mobility, nutrition, grip strength, energy as well as ADL, IADL, depression, ECOG-PS, and falls were significantly associated with final treatment recommendations (Table 2). Final treatment recommendations weren’t associated with Balducci’s classification (p = 0.58). In the multivariate analysis, a few of the frailty markers and ADL were significantly associated with final treatment recommendations (respectively *p*< 0.001 and *p* = 0.010) (Table 2).

**Table 2:** Univariate and multivariate logistic regression models showing the association between final treatment recommendations, CGA and Frailty markers.

		Univariate logistic regression				Multivariate logistic regression	
		Standard treatment with or without any changes	Modified treatment	Univariate OR [95%CI]	*p*	MultivariatOR [95%CI]	*p*-value
**Age (years)**		82.3±5.1	83.8 (±5.4)	0.9 [0.9;1.0]	0.08	-	-
**Sex**							
	Male	61(66.3%)	31 (33.7%)	1	0.093	-	-
	Female	68 (54.4%)	57 (45.6%)	0.6 [0.3;1.1]			
**Social support**							
	No	17 (63%)	10 (37.0%)	1	0.63	-	-
	Yes	107 (58.2%)	77 (41.8%)	0.8 [0.4;1.8]			
**Comorbidities ≥ 3**							
	No	109 (61.6%)	68 (38.4%)	1	0.21	-	-
	Yes	19 (50%)	19 (50%)	0.6 [0.3;1.3]			
**ADL**							
	Normal	92 (69.7%)	40 (30.3%)	1	<0.001*	1	0.010*
	Abnormal	36 (43.9%)	46 (56.1%)	0.3 [0.2;0.6]		0.4 [0.2 ; 0.8]	
**IADL**							
	Normal	57 (73.1%)	21 (26.9%)	1	0.002*	-	-
	Abnormal	71 (51.8%)	66 (48.2%)	0.4 [0.2; 0.7]			
**Cognitive impairment**							
	No	78 (61.4%)	49 (38.6%)	1	0.53	-	-
	Yes	48 (57.1%)	36 (42.9%)	0.8 [0.5;1.5]			
**BMI**		25.0 (±5.0)	24.5 (±5.4)	1.0 [0.9;1.1]	0.57	-	-
**Falls**							
	No	86 (65.2%)	46 (34.8%)	1	0.006*	-	-
	Yes	24 (43.6%)	31 (56.4%)	0.4 [0.2;0.8]			
**Depression**							
	No	89 (66.9%)	44 (33.1%)	0.5 [0.3; 0.9]	0.020*	-	-
	Yes	40 (50.0%)	40 (50.0%)	1			
**ECOG-PS**							
	< 2	43 (66.2%)	22 (33.8%)	1	0.010*	-	-
	≥ 2	10 (37.0%)	17 (63.0%)	0.3 [0.1;0.8]			
**Balducci’s Classification**							
	I	3 (60.0%)	2 (40.0%)	1	0.58	**-**	**-**
	II	119 (60.4%)	78 (39.6%)	1.1 [0.2;6.2]			
	III	7 (46.7%)	8 (53.3%)	0.6 [0.1;4.6]			
**Hearing deficit**							
	No	73 (65.2%)	39 (34.8%)	1	0.11	-	-
	Yes	49 (53.8%)	42 (46.2%)	0.6 [0.4;1.1]			
**Visual deficit**							
	No	78 (62.9%)	46 (37.1%)	1	0.38	-	-
	Yes	45 (56.3%)	35 (43.7%)	1.3 [0.7;2.3]			
**Numbers of frailty markers**							<0.0001*
	Not-frail: 0 marker (n = 15)	14 (93.3%)	1 (6.7%)	15.8 [1.9 ; 128.0]		21.8 [2.8;172.8]	0.004*
	Pre frail: 1–2 markers (n = 110)	79 (71.8%)	31 (28.2%)	3.2 [1.7 ; 6.1]		4.0 [2.2;7.2]	<0.0001*
	Frail: ≥ 3 markers (n = 92)	36 (39.1%)	56 (60.9%)	1	0.0001*	1	
**Grip strength**							
	Normal	94 (69.7%)	41 (30.4%)	1	< 0.0001*	-	-
	Abnormal	14 (28.6%)	35 (71.4%)	0.2 [0.1; 0.4]			
**Physical activity**							
	Normal	55 (80.9%)	13 (19.1%)	1	< 0.0001*	-	-
	Abnormal	63 (48.8%)	66 (51.2%)	0.3 [0.1;0.5]			
**Mobility**							
	Normal	35 (77.8%)	10 (22.2%)	1	0.002*	-	-
	Abnormal	80 (51.6%)	75 (48.4%)	0.3 [0.1;0.7]			
**Nutrition**							
	Normal	58 (69.0%)	26 (31.0%)	1	0.030*	-	-
	Abnormal	71 (54.2%)	60 (45.8%)	0.5 [0.3;0.9]			
**Energy**							
	Normal	93 (62.0%)	57 (38.0%)	1	0.036*	-	-
	Abnormal	7 (36.8%)	12 (63.2%)	0.4 [0.1;0.9]			

Abbreviations: CGA: Comprehensive Geriatric Assessment, OR: odds ratio, CI: Confidence Interval, ADL: Activities of Daily Living, IADL: Instrumental Activities of Daily Living, BMI: body mass index, ECOG–PS: Eastern Cooperative Oncology Group Scale of Performance Status

*: statistically significant

## Discussion

To our knowledge, this is the first study to address the association between frailty markers and treatment recommendations in older cancer patients. Frailty markers were highly prevalent. Almost 90% of patients presented with at least one frailty marker. We found a significant association between the number of frailty markers and ADL, and final treatment recommendations proposed after the 2^nd^ MDT. As the number of frailty markers increased, treatment recommendations were accordingly oriented toward modified treatment. On the other hand, the absence of frailty markers was associated with standard treatment. We observed although only 7% of our patients were considered as frail in Balbucci classification, we proposed for 40% a modified treatment.

Choosing the most sensitive tools to accurately assess health status is a major issue in geriatric oncology. The International Society of Geriatric Oncology (SIOG) and numerous literature reviews propose the use of a Comprehensive Geriatric Assessment (CGA) to determine optimal oncologic care on the basis of the patient’s health status rather than empirical evidence. However, several recent literature reviews have questioned the real value of the CGA in older cancer patients, as CGA seems to have a ceiling effect in detecting vulnerability in this population [7,8,9]. According to literature data, patients referred to oncology represent a population that differs from traditional geriatric patients: they have fewer comorbidities, fewer cognitive disorders, and good functional status at the time of diagnosis [37,38]. As in Chaibi’s and Caillet’s studies [11,14], we found a significant association with final treatment recommendations and ADL. However, our study found that abnormal ADL is observed in fewer than 30% of older cancer patients referred in oncology. A need for more sensitive tools is then indicated to identify patients who appear healthy but are vulnerable to complications in response to aggressive cancer treatments [8]. As patients get older, the risks of treatment toxicities increase. The result is then a narrowing of the therapeutic window between meaningful, positive effects and unacceptable side effects. Our results suggest that frailty markers may be highly helpful in this situation.

Several prospective studies in oncology have demonstrated the predictive value of frailty markers for treatment toxicities. In two studies of older patients with colon cancer, patients with at least three markers had higher risks of developing postoperative major complications [19] and early death [39,40]. In a study of almost four hundred cancer patients, Makary et al. [18] showed that preoperative frailty was associated with an increased risk for postoperative complications. Patients with two or three markers had two times higher odds (95% CI, 1.18–3.60) of developing complications, and patients with four or five markers had 2.5 times higher odds (95% CI, 1.12–5.77), in contrast to the patients with one or no markers. The presence of at least three markers independently predicted an increase in length of stay (p<0,001). Whatever the number of frailty markers, it appears that some markers have their own predictive value. Poor energy, abnormal nutrition and poor mobility were significantly predictive for early deaths [17,40,41]. Grip strength was also identified as an independent factor that predicted chemotoxicity [42] in older cancer patients and predicts adverse outcomes and postoperative morbi-mortality, regardless of age [43,44]. In a systematic review, Bohannon *et al*. concluded that grip strength is a predicting factor for mortality, disability, complications, and increased length of stay in middle-aged and older patients [45]. Today, it’s too premature to recommend systematic assessment of frailty markers in older cancer patients. But, regarding these encouraging studies, further ones are warranted to define if the use of frailty markers is the proper instrument to assess the risks of mortality and treatment complications in both simple and more accurate way compared to CGA [46]. We can also hypothesized a combination of predictors including frailty markers, some domains of CGA and cancer characteristics will probably be more useful clinically in order to capture complexity of older cancer patients as suggested by Bergman H. and collegues [47].

Our study presents several limitations. First, although realized in different hospitals, the geriatric assessment was performed by a single medical team. Therefore, the significant association observed in our study would have to be confirmed by similar studies conducted by other medical teams. Second, patients included in our study were referred by their specialists irrespectively of any screening tools; the geriatric status of patients not referred for CGA is unknown as other studies [48]. Our population is the one who really need to be assessed regarding the number of patient classified into groupe II or III of Balducci's classification. Two important strengths of this study are the use of validated self-report and performance tests and the high median age of our sample (median age: 83 years).

## Conclusion

In geriatric oncology, optimal management of older cancer patients is challenging, as the assessment of the underlying vulnerability guide**s** decision-making. Results of our study suggest that the use of frailty markers could help oncologists and geriatricians in their decision-making. Longitudinal studies are warranted to better determine their use in geriatric oncology.
